# Community perceptions of health accountability meetings with local politicians to improve healthcare quality: a qualitative study in Western Uganda

**DOI:** 10.1186/s12889-024-21025-3

**Published:** 2024-12-18

**Authors:** HaEun Lee, Neyat Fiseha, Jordan Bateisibwa, Cheryl A. Moyer, Joshua Greenberg, Elisa Maffioli

**Affiliations:** 1 Department of Systems, Populations, and Leadership , University of Michigan School of Nursing, Ann Arbor, Michigan USA; 2 University of Michigan Medical School, Ann Arbor, Michigan USA; 3 Progressive Health Partnership, Mbarara, Uganda; 4 Learning Health Sciences, Obstetrics and Gynecology, University of Michigan, Ann Arbor, Michigan USA; 5 Center for Global Health Equity, University of Michigan, Ann Arbor, Michigan USA; 6 Health Management and Policy School of Public Health, University of Michigan, Ann Arbor, MI USA

**Keywords:** Political accountability, Governance, Healthcare service, Meetings, Collective empowerment, Uganda

## Abstract

**Background:**

Lack of accountability within healthcare systems contributes to suboptimal healthcare quality and ultimately poor health outcomes, especially in low-income countries. In Uganda, our research team implemented a pilot project of quarterly health accountability meetings between community members and their local political leaders to discuss healthcare needs and strategies for quality improvement. In this study, we examine the community members’ understanding and perceptions of the health accountability meetings, as well as the perceived impact of the meetings on local healthcare services and community life.

**Methods:**

We conducted a total of 12 focus group discussions (FGDs), half with men and half with women, in November 2022 across six randomly chosen communities out of the ten communities where health accountability meetings were held. We audio taped, transcribed, and translated all FGDs into English. We collected data on demographics, understanding of the meetings, and perceived changes within healthcare services and the community from 111 participants. Two researchers analyzed the data using an inductive thematic approach, generating five themes.

**Results:**

We found the following themes: (1) increased inclusivity and promotion of bidirectional communication; (2) increased understanding of patient rights and practicing of collective empowerment by the community; (3) improved provider behavior; (4) enhanced relationships among politicians, community members, and healthcare providers; and (5) identified needs for future improvements.

**Conclusion:**

Through this qualitative study, we found that the community members perceived the accountability meetings as beneficial in improving the local healthcare services and community life. The study demonstrates the need to prioritize the voices of local communities in efforts to address the accountability gaps, as well as the potential for utilizing the relationship between community members and politicians to address accountability shortfalls in other governmental functions beyond healthcare.

**Trial registration:**

N/A.

**Supplementary Information:**

The online version contains supplementary material available at 10.1186/s12889-024-21025-3.

## Background

While a complex intersection of political, sociocultural, and economic factors contributes to poor quality health services in low-income countries (LICs), widespread accountability gaps in the health system also play a role in the delivery of suboptimal care [1]. Accountability includes the obligation to inform and explain actions or decisions to others, linking those who perform tasks to those who are affected by the tasks performed [2]. In the context of healthcare, lack of accountability can lead to problems such as health worker absenteeism, low health worker performance, poor facility infrastructure, diversion of medications and supplies, and out-of-pocket fees charged to patients [3, 4]. These challenges are particularly pronounced in Uganda, where decentralization reforms intended to improve governance have been undermined by weak oversight mechanisms, corruption, and resource constraints [5]. Moreover, social accountability mechanisms, such as community monitoring and citizen engagement, have shown promise in improving service delivery but are highly dependent on the countries’ capacities, information and range of different actors [6]. Addressing these accountability gaps is essential to enhancing healthcare quality and ensuring equitable access in LICs.

Many interventions that have aimed to mitigate accountability gaps have focused on the relationship between healthcare providers and community members who reside in a specific area where the services provided by these providers are often the only means of accessing formal healthcare. Researchers implemented a community-based monitoring of healthcare providers in Uganda, sharing information regarding local health outcomes and patients’ rights and facilitating meetings among healthcare providers and community members [7]. The intervention led to improved health worker performance, increased healthcare utilization, and improved child health outcomes [7]. Another team of researchers, evaluating the impact of information provision alone and with community meetings on healthcare provider behaviors and health outcomes in India, showed mixed results [8]. They found no impact on child mortality for both intervention arms but observed improvements in immunization, children’s nutritional status, and rates of institutional delivery, with marginally larger improvements when information provision and community meetings were implemented together [8]. While focusing on the healthcare provider-patient relationship has proven productive, researchers speculate that relying on the relationship between community members and healthcare providers may be insufficient [9, 10], since community members lack the power to impose formal sanctions on and rewards for healthcare providers.

In light of this observation, other researchers investigated the role of politicians in addressing these accountability gaps. In a recent study conducted in Uganda, researchers collaborated with the Ministry of Finance to explore the role of local politicians in exercising oversight and in holding the bureaucracy accountable to improve service delivery [11]. They provided local politicians with training about their responsibilities and rights, as well as quarterly financial information regarding politicians’ respective constituencies. The researchers found increased politician monitoring effort and improved stakeholders’ satisfaction with the quality of service delivery, but only in areas where the political leadership was not aligned with the dominant political party [11]. While the study did not focus on accountability for health services specifically, it demonstrated that, where the incentives are sufficiently high, local politicians can be empowered to reduce accountability gaps [9].

Addressing the health sector, Greenberg (2021) conducted a pilot study seeking to mitigate accountability gaps in health service provision by improving the politician-community relationship [12]. Unlike the relationship between healthcare providers and community members, where community members lack the power to directly hold healthcare providers accountable, politicians have a vested interest in addressing the healthcare needs of the community. This is because local politicians’ elections depend on the votes of community members, creating an incentive for them to prioritize healthcare quality [12]. In contrast to most interventions that relied on the healthcare provider-community relationship [9, 10], the pilot project involved local politicians who have the power to impose formal sanctions on and rewards for health providers.

In Uganda, local politicians can mitigate accountability gaps by monitoring service provision at local government health centers (HC). The government health service structure includes national and regional hospitals, with four levels of HCs [13]. HC I is the lowest level, comprising of community health workers referred to as Village Health Team members who link the community to higher levels of HCs. HC IIs serves about 5,000 people, managed by nurses and midwives, while and HC IIIs cater to approximately 20,000 people, providing outpatient care, immunization, antenatal care, vaginal and assisted deliveries. HC IVs serve around 100,000 people, offering inpatient care and surgeries [13]. Approximately 85% of Ugandans in the poorest two quintiles rely on government facilities, mainly at the HC II and at HC III levels [14]. Hence, these centers often serve as the only means to access health services for the most vulnerable Ugandans. Each HC III operates in a locality (either a subcounty, town council, or division) overseen by a locally elected politician, called the Local Council III (LCIII) Chairperson, who supervises health services [15].

Our research team conducted a pilot study that took the form of a stratified, cross-randomized controlled trial conducted in 20 localities containing HCIII’s in the Ankole Region of Uganda [12]. The primary purpose of the pilot study was to test two interventions aimed at improving the performance of local politicians in ensuring quality services at local health centers. The first intervention was a series of health accountability meetings between community members and the LCIII Chairperson to discuss health services and means for improvement. The second intervention was a health leadership program to equip LCIII Chairpersons with skills to better monitor and supervise local government health centers.

We held the health accountability meetings between Community members and LCIII Chairpersons on a quarterly basis to discuss healthcare quality and exchange regular feedback and reporting [12]. Public announcements, posters, community leaders, and healthcare providers encouraged community members to participate in the meetings. The structure of the meetings was standardized across communities and included: (1) welcoming participants and the LCIII Chairperson, (2) introducing the purpose of the meeting and establishing ground rules, (3) breaking into small groups to discuss concerns related to health services, (4) sharing written summaries from each group, (5) discussing any additional questions or comments, and (6) allowing the LCIII Chairperson to respond and to identify next steps to address concerns. Subsequent meetings allocated time for the local political leader to report on the progress from the previous meeting’s discussion.

While the pilot project demonstrated feasibility and scalability, it is unclear what the community members’ understanding, expectations, and outcomes of the health accountability meeting are. The key reasons for incorporation of community members’ participation into the accountability process is to empower the community members to influence the decisions made by the local government concerning healthcare resource use and service delivery. Therefore, it is critical to capture community members’ experiences and perceptions regarding the health accountability meetings. The qualitative study herein aims to understand community members’ perceptions – both men’s and women’s – of the health accountability meetings and meeting outcomes, specifically regarding the impact on local healthcare services and community life.

## Methods

### Sampling and recruitment

We randomly chose six communities out of the 10 communities from the pilot project that received the health accountability meetings. Greenberg (2021) includes the details of the selection process of the 10 communities elsewhere [12]. While we are primarily interested in the community’s perception of the health accountability meeting intervention, we sampled from the two of the four pilot trial arms that received the accountability meeting intervention (arm 1: accountability meeting only and arm 3: both accountability meeting and politician training). Each arm contained five communities. We randomly chose three communities from each of the two arms. This approach allowed us to further assess whether community perceptions differed between the two groups.

Our implementing partner, Progressive Health Partnership (PHP), worked closely with the Village Health Team (VHT) members in each community to recruit community members, both men and women, using a purposive sampling method. VHTs, also commonly known as community health workers, are trained individuals who work at the grassroot level to improve health outcomes in their communities. While they serve as liaison between healthcare providers and community members, they are also part of the community themselves, representing and advocating for the community’s perspectives. Since VHT members themselves attended the meetings and knew other community members who had attended the meetings, they recruited 10 to 12 participants for each focus group discussion (FGD) via phone. We conducted separate FGDs for men and women to reduce the possibility of women not speaking up in mixed-gender settings. The inclusion criteria were: (1) having attended at least one of the three health accountability meetings and (2) being 18 years or older.

### Data collection

We used a semi-structured focus group guide and conducted 12 FGDs in November 2022. We conducted the FGDs three to four months after the third and final health accountability meeting took place in August 2022. This timing provided participants the opportunity to experience and observe potential changes in local healthcare services and within their community.

Two Ugandan research assistants (RAs) fluent in both English and Runyankore, a local language widely used in the Ankole region, led each of the FGDs. The RAs completed a one-day training on focus group facilitation skills and procedures. Both RAs had extensive previous experience facilitating FGDs. FGDs were conducted during weekdays per participant convenience. Each FGD took approximately 60 to 90 min. RAs read the consent form, each of the survey questions, and all answer options for participants who were illiterate or with low literacy levels. First, the RAs read and collected the informed consent from each of the participants. Second, the RAs administered a brief demographic survey (Supplementary 1) with questions such as participant gender, age, marital status, number of accountability meetings attended, and number of visits to a HC in the past 6 months. Next, the RAs followed a semi-structured FGD guide (Supplementary 2) developed by the research team. The guide consisted of three main sections: (1) participants’ general understanding of the health accountability meeting; (2) changes, if any, as perceived by the participants, in HCIIIs; and (3) changes, if any, as perceived by the participants, in the community’s collective empowerment. With each section, the guide included prompts for the RAs to utilize as needed. Upon completion of the FGD, each participant received a bar of soap as a token of appreciation for their time and input. All FGDs were audio-recorded.

### Data analysis

Two additional RAs that did not facilitate the discussions and were not aware of the discussion content transcribed the audio recordings and then translated the transcriptions into English. The RAs reviewed each other’s work to ensure the quality of the transcriptions and translations. Then, two researchers (HL, NF) coded the translated data using an inductive thematic approach [16]. The two researchers independently developed the initial codes and then compared the codes for consistencies. Any identified inconsistencies were discussed until consensus was reached. The researchers and RAs who facilitated the FGDs then convened virtually to finalize the coding scheme, confirming the codes based on the data. Once the coding scheme was complete, the two researchers independently coded all the qualitative data. A researcher (HL) randomly selected a quarter of the transcripts (3 of the 12) to compare the two researchers’ codes, which matched at 82.5%. The two researchers further reviewed the codes to generate themes, checking for internal homogeneity to ensure similarity within each of the subthemes and for external heterogeneity to confirm distinctions between themes [16]. The qualitative data analysis was conducted with a mix of Nvivo, Microsoft Word, and Excel. Demographic data were collected on paper, entered into Microsoft Excel, and tabulated using Stata (17.0).

### Ethics

We obtained ethical approval for the research from the University of Michigan (#HUM00146736), Makerere University (#12.18.238), and the Uganda National Council for Science and Technology (#SS265ES).

## Results

### Characteristics of participants

Table 1 shows the demographic characteristics of the FGD participants. A total of 111 people, 54 (48.65%) men and 57 (51.35%) women, participated with an average of nine participants in each FGD. The average age of the participants was 49 years old, with the average age of men and women being 56 years old and 43 years old, respectively. Most participants were married/living with a partner (90.99%), with all (100%) of the men being married/living with a partner and 82.5% of the women being married/living with a partner. 70.27% of the participants had primary or lower level of education. On average, participants had 5 children, with 72% of the participants’ youngest child being 5 years or older. 95% of the FGD participants attended two or more meetings of the three total accountability meetings. On average, the participants visited the HC 2.7 times in the past six months.

**Table 1:** Descriptive analysis of the focus group discussion participants

	Total	Men	Women
	111 (100)	54 (48.65)	57 (51.35)
Locality *n* (%)			
Kagango (M)	20 (18.02)	11 (20.37)	9 (15.79)
Ruhumuro (M)	17 (15.32)	9 (16.67)	8 (14.04)
Rwoburunga (M)	20 (18.02)	9 (16.67)	11 (19.30)
Kabira (B)	17 (15.32)	8 (14.81)	9 (15.79)
Kibatsi (B)	19 (17.12)	9 (16.67)	10 (17.54)
Kaberebere (B)	18 (16.22)	8 (14.81)	10 (17.54)
Intervention arm *n* (%)			
Meeting only	57 (51.35)	29 (53.70)	28 (49.12)
Meeting and politician training	54 (48.65)	25 (46.30)	29 (50.88)
Age, mean (SD)	49.3 (15.00)	56.38 (13.09)	42.77 (13.71)
Relationship Status *n* (%)			
Married/living with partner	101 (90.99)	54 (100)	47 (82.46)
Divorced/separated	1 (0.90)	-	1 (1.75)
Widowed	7 (6.31)	-	7 (12.28)
Single	2 (1.80)	-	2 (3.51)
Education level *n* (%)			
Never went to school	9 (8.11)	3 (5.56)	6 (10.53)
Primary	69 (62.16)	34 (62.96)	35 (61.40)
Secondary	22 (19.82)	9 (16.67)	13 (22.81)
Tertiary or University	11 (9.91)	8 (14.81)	3 (5.26)
Number of living children, mean (SD)	5.56 (3.33)	6.67 (3.37)	4.52 (2.96)
Youngest child *n* (%)			
Younger than 6 months	6 (5.41)	2 (3.70)	4 (7.02)
Between 6 months − 1 year	3 (2.70)	-	3 (5.26)
Older than 1 years old	22 (19.82)	11 (20.37)	11 (19.30)
5 years or older	80 (72.07)	41 (75.93)	39 (68.42)
Number of accountability meeting attended *n* (%)			
1	6 (5.41)	3 (5.56)	3 (5.26)
2	63 (56.76)	25 (46.30)	38 (66.67)
3	39 (35.14)	23 (42.59)	16 (28.07)
Number of visits to health facility in the past 6 months mean (SD)	2.74 (2.29)	2.96 (2.62)	2.54 (1.92)

(M): Meeting only

(B): Both interventions

(SD): Standard deviation

### Themes

The main themes include (1) increased inclusivity and promotion of bidirectional communication; (2) increased understanding of patient rights and practicing of collective empowerment by the community; (3) improved provider behavior; (4) enhanced relationships among politicians, community members, and healthcare providers; and (5) identified needs for future improvements.

### Increased inclusivity and promotion of bidirectional communication

The FGD participants highlighted that, unlike other community meetings, the health accountability meetings were *inclusive* and allowed *bidirectional communication.* Participants pointed out that other meetings are often attended by community elites and are by invitation only. However, the health accountability meeting attendees were diverse and representative of their communities, with men, women, community leaders, and non-elite lay people, community members not participating in any leadership position, attending. They also mentioned that the meetings were facilitated in a manner that encouraged everyone to share.

*“In some of the [health accountability] meetings that I attended*,* they used to give a person a chance to ask and be listened to very well. In fact*,* the rest of us would be very attentive to listen to whatever he or she is talking about…every person in the meeting is free to speak out his or her idea and at the end of it all*,* they would be very important.”* (M7, Ruhumuro).

Furthermore, the participants reiterated that the health accountability meetings allowed bidirectional communication. Other community meetings providing education about health or household development are often unidirectional, with information passed from a few experts to the rest of the meeting attendees. However, during the health accountability meeting, the community members had an opportunity to converse with the politician rather than solely being on the receiving end of education or announcements.

*“It is from such meetings where community members get an opportunity to ask him [the LCIII Chairperson] several questions*,* and he also responds to them very well accordingly. He usually explains very well his duties and responsibilities.”* (W1, Ruhumuro).

### Increased understanding of patient rights and practicing of collective empowerment by the community

Participants stated that through the health accountability meetings they have gained a better *understanding of their rights* as patients regarding the services available and the quality of services they should expect to receive.

*“We learned from our leaders about the issues of privacy and confidentiality. You may find that your husband is HIV-positive*,* and you are not…You need to share your health condition with only the healthcare providers*,* and they cannot share it with other people. That was a very good thing we learned about healthcare providers to keep our health issues confidential.”* (W7, Kibatsi).

Additionally, participants also mentioned that they gained a sense of *collective empowerment*. After the meetings intervention, they felt more empowered when interacting with healthcare providers. If they were being mistreated by healthcare providers, the community members knew that they had access to the politician to further address the issue. Furthermore, the participants noted that witnessing specific changes after the discussions with the politician gave them a sense of confidence that together, as a community, they can make positive changes for their HCIIIs.

*“I learned that whenever there is a concern affecting people in the community*,* if it is shared and discussed in such meeting*,* it can be understood*,* addressed*,* and provided with a solution.”* (W2, Kaberebere).

*“…community members can influence political leaders to address some of the challenges affecting the facility. As I speak*,* we have enough stocks of drugs at the health facility. In addition to that*,* they [politicians] were able to advocate for more health care providers at the facility and we no longer have challenges with inadequate staff at the facility. Healthcare providers are well mannered. They give us all the health services that we need.”* (W9, Kabira).

### Improved provider behaviors

Relatedly, the participants perceived that, following the health accountability meetings, healthcare providers’ attitudes improved, and community members received more *respectful care* from providers. Participants stated that, prior to the meetings intervention, women generally were afraid to receive reproductive health services from the health facility because they were often dismissed and treated disrespectfully. However, after the meetings intervention, providers treated patients with more respect, taking their time to explain and educate.

*“Healthcare providers never used to care about pregnant mothers*,* especially after giving birth. But now*,* after the meetings were conducted*,* every mother who is done with childbirth is being attended to and given all the care that she deserves. Even if they [the healthcare providers] get some challenges*,* they do their best to save the situation. So*,* there is great improvement in service delivery.”* (W1, Rwoburunga).

After the meetings, community members also observed *lower provider absenteeism*. While there was some variability based on the community and the healthcare provider, participants indicated that because of the accountability meetings, certain healthcare providers known to act rudely and to flout normal working hours were transferred to different facilities. Since then, most healthcare providers were present at facilities and the community members could rely on finding a provider to receive care when they visited health facilities.

*“…We had challenges during the weekend in accessing healthcare*,* but after [the health accountability meeting]*,* you can get health workers available over the weekend. In fact*,* people from the village are assured of finding a health worker at the facility in case they are sick. So*,* the services have improved*,* and it is not like before.”* (M1, Kangango).

### Enhanced relationships among politicians, community members, and healthcare providers

During the meetings, community members gained a better understanding of providers’ challenges at work. While the accountability meetings primarily focused on the political leaders and the community members, a few of the political leaders further reached out to healthcare providers to attend the meetings as well. Hence, from the healthcare providers who attended the meetings as well as the politicians’ feedback, community members became more sympathetic toward healthcare providers. As a result, noteworthy improvement in the *relationships between community members and healthcare providers* were mentioned.

*“You find people saying whatever they want*,* sometimes that healthcare providers refuse to give people medicine intentionally when they are sick*,* but when the facility is supplied with few medicines*,* it gets finished so fast. But when healthcare providers and political leaders explain to us [the community members]*,* we also understand their position and we take it easy*,*”* (W7, Kibatsi).

Similarly, the FGD participants expressed a sense of understanding of the politicians’ roles and indicated that their *relationship with the politicians* has improved over time. Several participants mentioned that politicians play a role akin to the “household head,” by trying to listen to and address different people’s needs. They acknowledged that politicians cannot address every issue present at the health centers, and that it is time-consuming to escalate certain issues to higher-level politicians to bring change. The participants also recognized the meetings as a forum to hold politicians accountable and monitor their performance. Many of the participants described fostering a sense of friendship with their healthcare providers and politicians.

*“Political leaders are our voice as members of the community that they lead*,* so that they can forward our concerns or our needs to the authorities in the government where we do not reach.”* (M6, Kangango).

### Identified needs for future improvements

While the accountability meetings were perceived as beneficial overall, the participants also had several changes they hoped to see in the future. First, participants recommended that some type of *incentive* (e.g., transportation funds, refreshments) be provided at the meeting. Many participants travel and typically walk long distances to attend meetings, and because the meetings ensure everyone is heard, they tend to be lengthy. Participants reported that the time-consuming nature of these meetings led to lost income. Hence, the participants recommended that some food or transportation funds be provided.

*“To speak the truth*,* some people stay behind in the village and do not want to engage in such meetings. Most of them have work to do*,* they do casual work to earn an income. That is one of the reasons that affects them from attending such meetings. But if people know that when they come and they will get something*,* they will be motivated to come.”* (W6, Kabira).

Additionally, despite the designated time for politicians to report on their progress, the participants wished for *more thorough feedback* from the politicians regarding the actions they have taken based on the prior meetings’ discussions. They found that the feedback was limited and often felt that some of the same issues were being discussed in subsequent meetings with minimal improvements or action taken.

*“…I wish we would have other meetings to talk about the outcomes of the previous meetings or what was done and then we compare. When that is done*,* people will be motivated because they are sure what we discussed and what [we] shall discuss will be put under consideration as the items from the previous meetings were done.”* (M1, Kibatsi).

For all the 5 themes described above, we did not find meaningful differences in the discussion content between the two intervention arms or between the two genders of the participants.

## Discussion

We explored the perceptions of local Ugandan communities of health accountability meetings, an intervention implemented to provide a platform for local community members to express their healthcare-related concerns and to monitor politician performance on an ongoing basis. FGD participants stated that the health accountability meetings were unique compared to other community-based interventions and meetings in that they were inclusive and fostered bidirectional communication. These features offer important lessons for the design of community-based interventions, as it is important to consider characteristics such as gender and age as well as the participants’ roles within their communities to ensure that no sub-group dominates the representation and conversation [17]. The pilot data also showed that less powerful groups — such as women and non-elite community members — demonstrated noteworthy participation, as measured by both meeting attendance and discussion involvement [12]. Furthermore, the format in which the meeting participants were broken up into smaller groups and the group’s concerns were collected and shared allowed participants with different comfort levels in public speaking to be included in the meeting discussion. The bidirectional communication between politicians and community members is also rather uncommon, with education and announcements often being delivered by politicians to community members in a unidirectional matter.

In addition, we found that community members learned about their rights as patients and expanded their sense of collective empowerment. The Patients’ Rights Charter, which has been adopted by most countries across the globe, indicates that patients are expected to be aware of their rights and responsibilities to help encourage rational and ethical medical practices and to improve health outcomes [18]. Despite Uganda implementing the Patients’ Rights Charter in 2009, many patients still are not aware of their rights [18, 19]. Considering that patients’ understanding of their rights is critical to the quality of services they receive [20], it is noteworthy that the meeting intervention increased participants’ awareness of their rights as patients. However, as information delivery about patients’ rights does not always lead to positive healthcare utilization and outcomes, this observation must be interpreted carefully.

Furthermore, our findings illustrated that meeting participants gained collective empowerment in the context of healthcare, feeling a sense of competency and control over the decisions made regarding their healthcare services. This sense of agency evoked amongst community members is particularly salient given Uganda’s effort to decentralize government service provision. Allowing local community members to exercise influence over the decisions made by local government shifts the responsibilities for service delivery from central to local government for better use of resources [21]. Hence, the health accountability meetings align with the national drive toward citizen-led advocacy to ultimately stimulate government responses to improve health service delivery [22].

Additionally, participants perceived improvement in respectful care and provider absenteeism following the implementation of the health accountability meetings. Unfortunately, disrespectful and abusive care practices have long been identified as a substantial barrier to accessing care, in turn leading to poor health outcomes. Interventions have focused on sensitization programs for healthcare providers, education and empowerment programs for patients, and the enactment of policies and guidelines [23]. Health accountability meetings are an innovative intervention to enhance respectful care by empowering both community members and their political leaders. The meetings may also have crucial effects on health by addressing provider absenteeism; for example, a longitudinal analysis study in Uganda found that health worker absenteeism reduces the odds of a patient seeking care at public health facilities and receiving malaria testing, and increases the odds of paying out of pocket fees for treatment, presumably by sending patients to private facilities [24]. When patients regularly find providers absent, they stop seeking services, assuming that the providers will not be present to provide care. Hence, participants’ perception of improved respectful care and provider attendance may lead to improved health-seeking behavior. Nonetheless, if resources such as medications and testing equipment are unavailable, the continuum of care will remain disrupted, with patients ultimately not receiving quality care [25]. Therefore, while it is important to address provider attitude and absenteeism, adequate resources need to be provided to ensure overall access to and utilization of healthcare services.

Community members in this study perceived improved relationships between themselves, their providers, and their elected politicians, largely due to shared understanding of each other’s experiences and perspectives. A study conducted in Uganda exploring the reasons for absenteeism among health workers identified issues such as transportation barriers, personal/family-related problems, and delayed payment from the government [26]. The meeting intervention enabled community members to learn about the healthcare providers’ life circumstances, allowing the community members to cultivate a more forgiving attitude and ultimately an improved relationship with their providers. At the same time, more frequent contact and access to the politicians strengthened the community members’ relationship with politicians. Given that low expectations of government responsiveness are often a barrier to community-based accountability interventions [27, 28], it is noteworthy that the meeting intervention functioned as a platform for citizens to observe politician actions and listen to their points of view. In turn, this opportunity for exchange improved the community members’ confidence in the politicians’ ability to improve healthcare services.

Finally, participants suggested incentives for participation and more in-depth feedback from the politicians as improvements for future health accountability meetings. While incentives are ordinarily considered an ethical means to value participants’ time and effort [17], the provision of incentives may also come with pitfalls. In the context of civic engagement programs — in which a major goal is to stimulate a sense of civic responsibility on the part of citizens — incentives could have counterproductive effects by conditioning community members to expect personal rewards for their involvement in local governance and affairs. Similarly, for a program that ultimately might be integrated into regular community life, the provision of incentives would create meaningful budgetary implications and could potentially undermine its sustainability. In the end, careful determination of both the type and level of incentives, if any, will likely be critical to promoting pro-social behavior and to ensuring both the near- and long-term success of the health accountability meetings [29]. Furthermore, despite the pilot project not providing any incentives or transportation for participants, each meeting had high participation rate, suggesting the potential for community engagement and ownership when the purpose of accountability meetings is effectively communicated and has community member buy-in [12]. In regard to the suggestion for greater politician feedback, the pilot project protocol contained dedicated time for the politicians to share their progress and actions. However, the finding that participants desire longer dedicated time for politician feedback and follow-up will inform potential revisions to the intervention design.

Overall, few interventions focus on providing a platform between local politicians and community members to enhance healthcare accountability. Health accountability meetings may be a promising intervention with unique characteristics and perceived improvements in local healthcare services and community life.

## Limitations

This study has several limitations. First, as is true for all qualitative research, these findings cannot be generalized to a larger population as would be possible with quantitative data collected from a large, randomly selected sample. However, because the selection criteria included having attended at least one health accountability meeting across 6 communities and included both male and female genders, we believe the data captured a wide range of participants’ perceptions about the meetings and their perceived impact on healthcare services and community life. Second, social desirability bias may exist due to the facilitators’ affiliation with the local non-governmental organization, PHP, since PHP implemented the interventions and the FGD participants may have wanted the accountability meeting intervention to continue.Furthermore, we did not include any participants who had not participated in the meeting intervention, potentially excluding their perspectives on reasons why they decided not to participate or whether they also noticed any recent changes in the quality of healthcare services. However, feedback included both positive and negative issues, suggesting that social desirability bias was not a critical factor in our findings. Despite these limitations, this study adds important insights regarding participants’ understanding and perceptions of health accountability meetings as well as the intervention’s perceived impact on local healthcare services and community life.

## Conclusion

Accountability gaps in the healthcare system contribute to poor population health across many LICs. Hence, understanding the perceptions of communities around health accountability meetings between community members and politicians has important implications for the future design and scale-up of such interventions. Through this study, we demonstrated the need to prioritize the voices of local communities in efforts to address health service accountability gaps. Utilizing and leveraging the relationship between community members and politicians to address accountability gaps can be applied to other governmental functions beyond healthcare systems. Future research should further investigate the extent to which similar accountability meetings between community members and politicians can be used for other governmental functions in different LICs.

## Electronic supplementary material

Below is the link to the electronic supplementary material.

Supplementary Material 1

## Data Availability

The data that support the finding of this study are available from the corresponding author upon reasonable request.

## Declarations

### Positionality statement

In this study, the authors’ diverse backgrounds and roles significantly shaped the research process and its outcomes. JB, a Ugandan and co-founder of PHP, brought invaluable local insights and a nuanced understanding of the context, which were essential for the design and implementation of the study. JG, an economist and physician, is both a co-founder and the current director of PHP, overseeing all research activities and conducting the pilot intervention. As a permanent resident of Uganda, JG’s experience in the local context greatly informed the study. HL, an early-career professor, co-led the project with JB, contributing to both the design and execution phases. EM and CAM, senior professors, provided extensive experience and oversight, ensuring the study’s rigor and academic validity. The two Ugandan research assistants, CA and AT, were offered authorship positions but declined, as publication was not of interest to them. However, both CA and AT played a key role in facilitating the FGDs and collaborating with HL and NF to verify the coding process and the thematic analysis. GN and PG, both Ugandan research assistants, also contributed to translating and verifying all FGD transcripts, though their contribution did not meet authorship criteria. NF, a medical student, brought fresh perspectives and made important contributions to data analysis, interpretation, and manuscript development. Our varying levels of experience and expertise influenced our approach to the study and shaped our interpretations of the data. While HL, JG, and JB possess extensive experience working in Uganda, the senior perspectives of EM and CAM added a layer of academic robustness. The collaborative nature of our team enabled a comprehensive analysis, although it is important to acknowledge that our individual perspectives may have introduced different biases and interpretations.

## Abbreviations

LICs: Low-income countries
HC: Health center
LC III: Local council III
PHP: Progressive health partnership
VHT: Village health team
FGD: Focus group discussion
RA: Research assistant
