# Supplementary material for: Community perceptions of health accountability meetings with local politicians to improve healthcare quality: a qualitative study in Western Uganda
Source: BMC Public Health. 2024 Dec 18;24:3526. doi: 10.1186/s12889-024-21025-3 (PMC11656907; doi:10.1186/s12889-024-21025-3)
Supplement: Supplementary file 1 — Supplementary Material 1 [file 12889_2024_21025_MOESM1_ESM.docx]

*Supplementary 1.* Demographic survey for focus group discussion participants.

Date:

DD/MM/YYYY

Demographic Survey: Focus group discussion Participants

1. What is your gender (circle below)?
   1. Man
   2. Woman
2. How old are you? ____________
3. Relationship status
   1. Married/living with partner
   2. Divorced/separated
   3. Widowed
   4. Single
4. What is your education level (circle below)?
   1. Never went to school
   2. Primary
   3. Secondary
   4. Tertiary or University
5. How many living children do you have? ____________
6. How old is your youngest child?
   1. Younger than 6 months
   2. Between 6 months – 1 years
   3. Older 1 years old – younger than 5 years old
   4. 5 years or older
7. How many health meetings have you attended so far? _______
8. How many times have you visited the health facility in the past 6 months because you or your family members needed health care service (NOT for meetings)? ________ times

*Supplementary 2.* Semi-structured focus group discussion guide.

Focus Group Discussion Script

**Introducing the team**

Hello everyone, thank you so much for agreeing to participate in this group discussion. My name is [NAME OF FACILITATOR] and I will be the facilitator of this discussion. [BRIEFLY INTRODUCE YOURSELF.]

This is [NAME OF RESEARCHER], a researcher working with the Progressive Health Partnership (PHP) team. [BRIEFLY INTRODUCE RESEARCHER.] While we talk, he/she is going to observe and take some notes.

Finally, this is [NAME OF TRANSLATOR], who is working as a translator. [BRIEFLY INTRODUCE TRANSLATOR.] He/she will be translating the discussion for [NAME OF RESEARCHER] so that he/she can understand what we are talking about.

**Go over Informed Consent forms**

Did you go over the Informed Consent forms? _____

Did all the participants sign the Informed Consent forms? _____

**Go over demographic survey questionnaires**

Did all the participants fill out the demographic survey questionnaires? _____

**Now we will start the recording and state:**

Today’s date is [DATE], we are at [LOCATION, DISTRICT/SUB-COUNTY/PARISH/VILLAGE], and we are conducting this FGD with [MEN/WOMEN].

We would like to emphasize that we are trying to understand more about the health meetings, and we are not working with the government or health facilities directly. We are interested in hearing about the things discussed during the meeting, and whether certain improvements were made based on the meeting discussions, but we are not here to deliver the message to the politicians or the healthcare providers.

Please remember that the content of today’s discussion will be recorded and should not be discussed with other people after the focus group discussion.

Tell me what these health meetings are like.

*Probe:*

1. Who attends these meetings?
2. Why do people attend these meetings?
3. What happens during these meetings?
4. What is different about the health meetings compared to other types of community meetings?
5. Did the meeting meet your expectations? In what way? Can you say more?

Since the health meetings began, what changes, if any, have you seen in your community’s health facility?

Tell us about the changes, if any, in the way the community accesses healthcare services. Can you say more about if/how that has been affected by the health meetings?

Tell us about the changes, if any, in the way the community interacts with healthcare providers. Can you say more about if/how that has been affected by the health meetings?

Do you think the health meeting with the political leaders is effective in improving health service quality? Tell us more about why you think it is or isn’t. What other ways can health service quality be improved?

Since the health meetings began, what changes, if any, have you seen in your community’s ability to hold your leaders accountable? Can you say more about if/how that has been affected by the health meetings?

Since the health meetings began, what changes, if any, have you seen in your community’s ability to influence political decisions? Can you say more about if/how that has been affected by the health meetings?

Since the health meetings began, what changes, if any, have you seen in your community’s ability to make changes to improve the community? Can you say more about if/how that has been affected by the health meetings?

Since the health meetings began, what changes, if any, have you seen in the way people share their thoughts and opinions? Is everyone who wants to share their thoughts during the health meeting able to do so? Do you think everyone’s thoughts and opinions are equally valued?

Are there any other changes in your life or the community’s life that may have been affected by the health meetings?

What recommendations do you have to make the health meetings better?

Is there anything else about the meeting you want to talk about?

Thank you so much for your time. We have learned much from you, and we appreciate you sharing your thoughts with us. Please remember that the content of today’s discussion should not be discussed with other people.

**Stop recording.**
